# Trends in *Genomics & Informatics*: a statistical review of publications from 2003 to 2018 focusing on the most-studied genes and document clusters

**DOI:** 10.5808/GI.2019.17.3.e25

**Published:** 2019-09-27

**Authors:** Ji-Hyeon Kim, Hee-Jo Nam, Hyun-Seok Park

**Affiliations:** 1 Bioinformatics Laboratory, ELTEC College of Engineering, Ewha Womans University, Seoul 03760, Korea; 2 Center for Convergence Research of Advanced Technologies, Ewha Womans University, Seoul 03760, Korea

**Keywords:** document clustering, genes, shallow neural network, word cloud

## Abstract

*Genomics & Informatics* (NLM title abbreviation: *Genomics Inform*) is the official journal of the Korea Genome Organization. Herein, we conduct a statistical analysis of the publications of *Genomics & Informatics* over the 16 years since its inception, with a particular focus on issues relating to article categories, word clouds, and the most-studied genes, drawing on recent reviews of the use of word frequencies in journal articles. Trends in the studies published in *Genomics & Informatics* are discussed both individually and collectively.

## Introduction

*Genomics & Informatics* is the official journal of the Korea Genome Organization. The prototype version of the full-text corpus of *Genomics & Informatics* has recently been archived in the GitHub repository as GNI version 1.0 [1,2]. As of July, 2018, 499 part-of-speech–tagged full-text articles are available as a corpus resource. Now that the prototype GNI corpus has been constructed, we can obtain basic descriptive statistics. The most basic statistical measure is a frequency count, a simple tallying of the number of instances of something that occurs in a corpus [3].

Ensuring the validity of statistical conclusions involves using adequate sampling procedures and reliable measurement procedures. Issues of validity and reliability occur when the sample size of the study is too small given other factors. Still, the publications in *Genomics & Informatics* contain a substantial subset of scientific knowledge. Analyzing data from publication databases helps us understand how this knowledge is obtained and how it changes over time. For example, by comparing the empirical data on the popularity of genes analyzed in each volume, we might detect noteworthy publication patterns of a journal.

In this study, we present the temporal dynamics of the most-studied genes as a reflection of the scientific content of *Genomics & Informatics*. We also discuss article categories and present word clouds of articles published in *Genomics & Informatics*, using a shallow neural network and K-means clustering.

## The Most-Studied Genes in *Genomics & Informatics*

The biological literature is characterized by a heavy use of domain-specific terminology. In this analysis, our data set consisted of references to numerous genes in the 499 publications listed in *Genomics & Informatics*. Thus, the frequency of gene names is an excellent measure of trends in the academic papers published in this journal.

Initially, we extracted all papers tagged as describing the structure, function, or location of a human gene or the protein it encodes. Sorting through the records, we compiled a list of the most-studied genes in the journal. This list shows trends in research, revealing how concerns about specific diseases or health issues have shifted research priorities in the academic community towards the genes underlying these conditions.

**Fig. 1.**
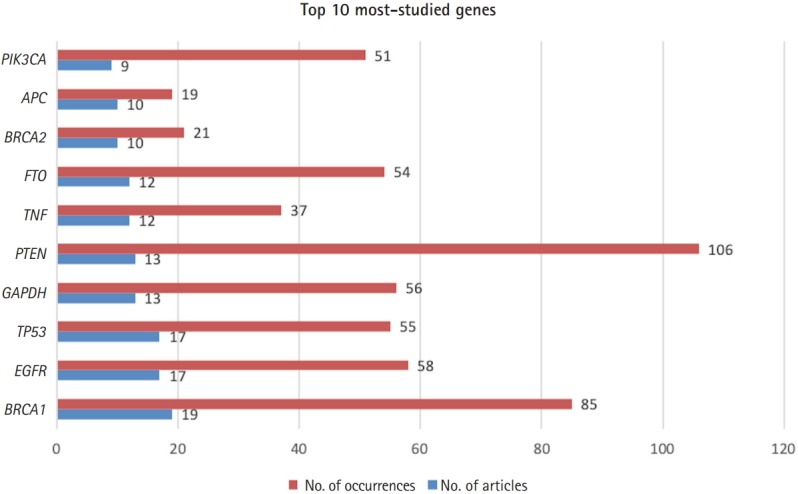
The top 10 most-studied human genes in *Genomics & Informatics*.

**Fig. 2.**
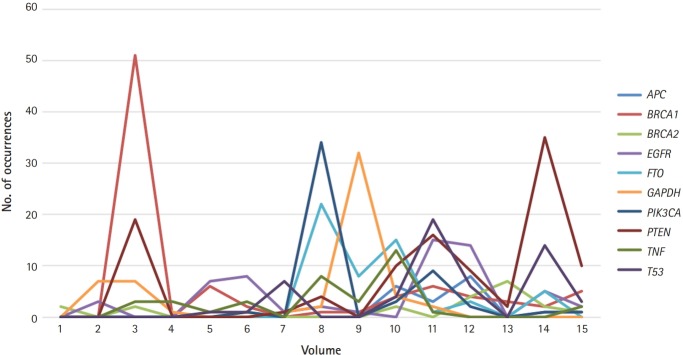
The temporal dynamics of the top 10 most-studied genes in *Genomics & Informatics: APC* (blue), *BRCA1* (red), *BRCA2* (green), *EGFR* (purple), *FTO* (light blue), *GAPDH* (orange), *PIK3CA* (dark blue), *PTEN* (dark red), *TNF* (dark green), and *TP53* (dark purple).

Fig. 1 shows the top 10 genes studied in *Genomics & Informatics*: *EGFR, BRCA1, TP53, PIK3CA, BRCA2, PTEN, GAPDH, TNF, FTO*, and *APC*. The list is ranked based on how many papers mentioned each gene name. For example, *BRCA1* appeared 85 times in 19 different publications, according to Fig. 1. Considering that many genes have only appeared once in the journal, these remarkable frequency differences may reflect differences in the importance of genes. In line with this reasoning, the most-studied human genes are related to human diseases. Almost all the most-studied genes are highly related to cancer, with the exception of *GAPDH*, a housekeeping gene. For example, the involvement of p110α, which is encoded by *PIK3CA*, in human cancer has been hypothesized since 1995, and *PIK3CA* started to appear in later volumes of the journal [4]. *TP53* is a well-known tumor suppressor that is widely known to be mutated in roughly half of all human cancers. *BRCA* mutations, in either the *BRCA1* or *BRCA2* gene, are also a well-known category of mutations, as women with harmful mutations in either *BRCA1* or *BRCA2* are known to have a risk of breast or ovarian cancer that is roughly five to 30 times the normal risk [5]. Fig. 2 shows the temporal dynamics of these top 10 genes over the last 16 years.

Compared with the list of the top 10 most popular genes in recent publications in Nature News (*TP53, TNF, EGFR, VEGFA, APOE, IL6, TGFB1, MTHFR, ESR1*, and *AKT1*) [6], the top 10 list of *Genomics & Informatics* shares several gene names. The observed dynamics may result from a simple process—namely, authors naturally publish on genes that have already appeared in other publications. This might be a rewarding strategy for authors, because there is a positive correlation between the frequency of a gene in scientific publications and the impact of related publications as assessed through journal metrics.

We also surveyed genes and proteins of non-human species. The list of the most frequent organism names appearing in *Genomics & Informatics* is as follows: *Escherichia coli* (218 times), *Saccharomyces cerevisiae* (73 times), *Caenorhabditis elegans* (37 times), *Acinetobacter baumannii* (23 times), *Drosophila melanogaster* (18 times), *Xenopus tropicalis* (15 times), *Pseudomonas aeruginosa* (15 times), *Sus scrofa* (14 times), *Oryza sativa* (13 times), *Bos taurus* (12 times), and *Arabidopsis thaliana* (12 times). Other species that appeared in the journal included: *Hepacivirus C, Rattus norvegicus, Chlamydomonas reinhardtii, Mus musculus, Schizosaccharomyces pombe, Danio rerio, Mycoplasma pneumoniae, Takifugu rubripes, Dictyostelium discoideum, Xenopus laevis, Plasmodium falciparum, Zea mays*, and many others.

However, it is less clear how the genes of these species were chosen by authors, and no clear relationship was observed between the popularity of a gene from non-human species and its importance to cellular processes. This indicates that in the case of species other than humans, the emergence of highly popular genes is not necessarily driven by importance alone, but also by other mechanisms, such as conventions and the individual author’s research field.

## Document Clustering

Another important measure to show the advancements and trends of *Genomics & Informatics* is classifying the documents into appropriate categories. By classifying articles in *Genomics & Informatics*, we aimed to assign one or more classes or categories to a document, making it easier to manage and sort. As an interdisciplinary scientific journal, *Genomics & Informatics* combines biology, computer science, information engineering, mathematics, medicine, and statistics to analyze and interpret biological data. Thus, the research articles of *Genomics & Informatics* may be classified into different groups based on specialty.

Document clustering involves the use and extraction of descriptors, which are sets of words that describe the content within the cluster. We chose K-means clustering, where the K-means process initializes with a pre-determined number of clusters [7,8]. The mean of the clustered observations is calculated and used as the new cluster centroid, in an iterative process until the algorithm reaches convergence.

We also used term frequency–inverse document frequency (TF-IDF) [9] to give different weighting to words based on their importance to a document in the journal. The TF-IDF weighting for a word increases with the number of times the word appears in the document, but decreases based on how frequently the word appears in the entire document set.

**Fig. 3.**
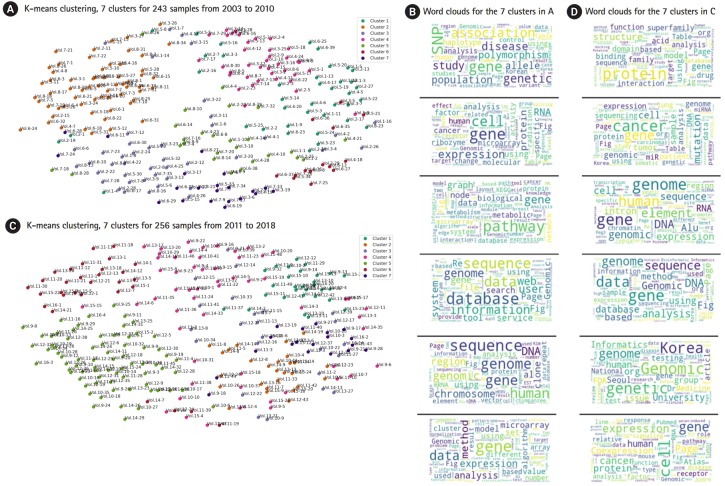
K-means clustering: comparison of seven clusters for 243 samples from 2003 to 2010 (A, B) with 7 clusters for 256 samples from 2011 to 2018 (C, D). Each dot represents a paper. For example, ‘Vol 13-27’ refers to paper no. 27 in volume 13.

Fig. 3A shows K-means clustering, with seven clusters for 243 articles published during the period from 2003 to 2010, and the data are displayed on a two-dimensional space. This reflects some degree of indexing and sorting of each cluster to identify the top n words that are nearest to the cluster centroid, which give a good sense of the main topic of the cluster. Publications in cluster 1 are presumed to belong to association studies (keywords: *SNPs, polymorphism*, and *association*). Publications in cluster 2 are presumed to belong to microarray and expression studies (keywords: *cell, expression, RNA*, and *proteins*). Likewise, cluster 3 is presumed to be related to pathway studies (keywords: *pathways, metabolic, network*, and *visualization*). Cluster 4 is assumed to be related to databases and computational methods (keywords: *databases, sequence, web*, and *search*). Cluster 5 seems to be related to studies of protein interaction (keywords: *protein, domains*, and *interaction*). The keywords for cluster 6 were *clone, human, chromosomes*, and *region*. The keywords for cluster 7 were *microarrays, expression, clustering*, and *normal*.

Fig. 3C shows K-means clustering, with seven clusters for 256 articles published during the period from 2011 to 2018, and the data are displayed on a two-dimensional space. The keywords of each cluster were slightly different from the version in Fig. 3A, which contained information for earlier articles. The keywords for cluster 1 were *model, protein structures, interaction*, and *prediction*. The keywords for cluster 2 were *cancer, mutations, tumor, samples*, and *cell*. The keywords for cluster 3 were *elements, transcription, expression*, and *DNA*. The keywords for cluster 4 were *DNA, database*, and *species*. The keywords for cluster 5 were SNPs, association, population, and genotype. The keywords for cluster 6 were *groups, review*, and *medicine*, and the keywords for cluster 7 were *expression, cancer*, and *protein activation*. It is noticeable that in later volumes, the keywords *medicine* and *cancer* appear more often. This coincides with the result shown in Fig. 2, where cancer-related genes appeared more often in later volumes of the journal.

There were some overlapping keywords between different clusters. For example, *microarray* appeared in both cluster 2 and 7 in Fig. 3A. Likewise, the keyword *cancer* appeared in both cluster 2 and cluster 7 in Fig. 3C. It is a common occurrence that search terms containing one or more same words belong to different topic groups because of the complexity of natural language. The process of identifying main topics through clustering requires some human judgment. This is one of the drawbacks of clustering, and it is beyond the scope of this paper.

Once we have tokenized data, a basic analysis that is commonly performed is counting tokens and their distribution in a document or set of documents. From the frequency distribution, we can generate a word cloud to obtain an intuitive visualization of the words used in the text. Finally, Fig. 3B and 3D shows some tag clouds for each cluster in Fig. 3A, and 3C, respectively [10,11]. A tag cloud is a visual representation of text data, typically used to depict keyword metadata, where the importance of each tag is shown with font size or color.

## Summary

We analyzed developments in the reporting of research in 499 articles published in *Genomics & Informatics* from 2003 to 2018. We discussed several issues relating to article categories, word clouds, and the most studied genes. The frequency distribution of genes discussed in *Genomics & Informatics* resembles a power law, as a few highly popular genes were found to dominate the literature. We also categorized the published articles using interdisciplinary terminology.

## References

[1] (2018). Genomics and Informatics archives. https://genominfo.org/articles/archive.php.

[2] Oh SY, Kim JH, Kim SJ, Nam HJ, Park HS (2018). GNI Corpus version 1.0: annotated full-text corpus of Genomics & Informatics to support biomedical information extraction. Genomics Inform.

[3] Biber D, Conrad S, Reppen R (1998). Corpus Linguistics: Investigating Language Structure and Use.

[4] Hiles ID, Otsu M, Volinia S, Fry MJ, Gout I, Dhand R (1992). Phosphatidylinositol 3-kinase: structure and expression of the 110 kd catalytic subunit. Cell.

[5] National Cancer Institute (2014). BRCA1 and BRCA2: Cancer Risk and Genetic Testing.

[6] Dolgin E (2017). The most popular genes in the human genome. Nature.

[7] Forgy E (1965). Cluster analysis of multivariate data: efficiency versus interpretability of classifications. Biometrics.

[8] Lloyd S (1982). Least squares quantization in PCM. IEEE Trans Inf Theory.

[9] Leskovec J, Rajaraman A, Ullman JD (2011). Mining of Massive Datasets.

[10] Mikolov T, Chen K, Corrado G, Dean J (2013). Efficient estimation of word representations in vector space. https://arxiv.org/abs/1301.3781.

[11] Halvey MJ, Keane MT, Williamson C, Zurko ME, Patel-Schneider P, Shenoy P (2007). An assessment of tag presentation techniques.

